# Temperature Induced Syllable Breaking Unveils Nonlinearly Interacting Timescales in Birdsong Motor Pathway

**DOI:** 10.1371/journal.pone.0067814

**Published:** 2013-06-20

**Authors:** Matías A. Goldin, Leandro M. Alonso, Jorge A. Alliende, Franz Goller, Gabriel B. Mindlin

**Affiliations:** 1 Laboratorio de Sistemas Dinámicos, Departamento de Física, Facultad de Ciencias Exactas y Naturales, Universidad de Buenos Aires, Buenos Aires, Argentina; 2 Department of Biology, University of Utah, Salt Lake City, Utah, United States of America; Claremont Colleges, United States of America

## Abstract

The nature of telencephalic control over premotor and motor circuits is debated. Hypotheses range from complete usurping of downstream circuitry to highly interactive mechanisms of control. We show theoretically and experimentally, that telencephalic song motor control in canaries is consistent with a highly interactive strategy. As predicted from a theoretical model of respiratory control, mild cooling of a forebrain nucleus (HVC) led to song stretching, but further cooling caused progressive restructuring of song, consistent with the hypothesis that respiratory gestures are subharmonic responses to a timescale present in the output of HVC. This interaction between a life-sustaining motor function (respiration) and telencephalic song motor control suggests a more general mechanism of how nonlinear integration of evolutionarily new brain structures into existing circuitry gives rise to diverse, new behavior.

## Introduction

Complex behavior arises from motor instructions that are generated by interconnected networks of brain areas. Birdsong is a complex, learned behavior which is generated by a well-described network of forebrain nuclei (HVC, used as a proper name and the robust nucleus of the arcopallium, RA) that generates the coordinated motor patterns of muscles controlling respiration, the vocal organ and upper vocal tract structures [1]. However, it has been debated to what degree this telencephalic motor program contains direct instructions for detailed patterns such as the various timescales of the behavioral output [2]–[10]. One proposed model for the role of cortical structures in song motor control is that of interaction with the nonlinear network for respiration [2], [9], [11]. In canaries (*Serinus canaria*), this model can account for all respiratory patterns underlying song and interprets the diverse respiratory patterns of different syllable types as the outcome of a nonlinear interaction between input from the telencephalon (HVC, RA) and internal dynamics of the respiratory pattern generators. The air sac pressure patterns during song were found to be consistent with the shapes and rhythms of the subharmonic solutions of a driven nonlinear system [2], [12], [13] similar to systems used by engineers and physicists for exploring complex behavior and chaos [14], [15]. This interactive model is in clear contrast to one proposing more direct control by the telencephalic song control area HVC in the zebra finch (*Taeniopygia guttata*), such that all timescales present in the song arise directly from the output signal of this nucleus. Support for this model was derived from observations of sparsely coding output neurons in HVC [16], [17] as well as experiments in which song was stretched by cooling of HVC [3], [18].

To explore whether one general mechanism may account for these conflicting results, we use the interactive model to predict the effects of cooling on the respiratory patterns of song and then test these predictions experimentally by bilateral cooling of HVC in the canary. If the song system integrates timescales generated in different regions of the motor pathway, stretching of respiratory patterns and song should only occur in a range of temperatures where the naturally occurring locking regime between the telencephalic input and the respiratory pattern generating network takes place. For example if periodic instructions from HVC (forcing) are locked to the respiratory pattern of song in a 2∶1 ratio (i.e., the respiratory frequency is a subharmonic of the forcing frequency), and we expect cooling to change the forcing frequency, a change in this locking is predicted to occur if the forcing frequency changes beyond a certain value.

## Results

### Using a computational model to predict the effect of cooling

**Figure 1:**
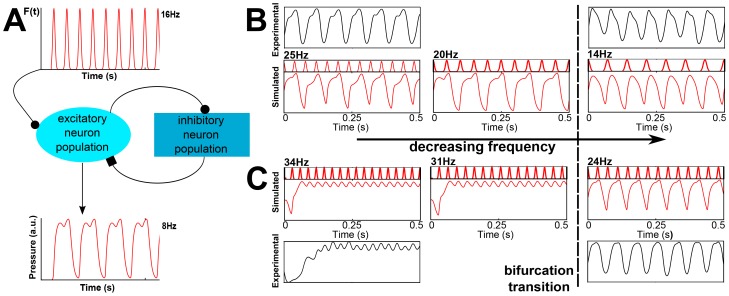
Dynamical model of motor pathway and simulations of canary pressure patterns during song. (A) Schematic of proposed connectivity of circuit components. At top, HVC average activity is modeled as a simple periodic instruction driving downstream neuron populations, one excitatory and one inhibitory (See Materials and Methods for complete model and parameters). Activity from the driven excitatory population is proposed to be proportional to the output motor instruction driving air sac pressure gestures. For the depicted input frequency from HVC (forcing frequency) of 16 Hz the output is a subharmonic frequency of 8 Hz. The air sac pressure range is from 0–1 in arbitrary units. (B-C) Simulations of pressure gestures that first stretch and then break (in red) compared to actual recorded data (black). Paired panels have simulated pressure gesture and the proposed driving, scaled for illustrating the locking behavior and subharmonicity. (B) Simulated syllables at 25 Hz, 20 Hz and 14 Hz of instruction frequency (with a changing amplitude 3.1, 3.0 and 2.9 to allow better matching with experimental patterns). The first two columns show locking at half the frequency of instruction (2∶1), whereas the last column shows locking at 1∶1. (C) Simulated syllables at 34 Hz, 31 Hz and 27 Hz instruction frequency (amplitude is 2.44). The first two columns are locked 1∶1 and in the last the frequency of instructions is halved (2∶1). Notice that only varying the driving frequency, (corresponding to the conjectured effect of slowing down the HVC activity), we can account for both effects of stretching and breaking. Subharmonicity appears for some frequency ranges. Dotted line corresponds to a bifurcation transition in the system, where output changes drastically as the forcing frequency changes gradually.

Our minimalistic dynamical model predicts that during initial cooling, stretching of syllables should occur, but that further cooling causes breaking of certain respiratory patterns. It assumes that two timescales interact nonlinearly. The first is associated with HVC activity, and although a number of timescales are expected to be present in HVC output, we will concentrate on the timescale associated with repeating syllables. For swamp sparrows (*Melospiza georgiana*), it has been reported that HVC projection neurons repeat their spiking pattern when a syllable is being repeated [19]. Although this has been reported for neurons projecting to area X, it is likely that RA-projecting neurons in HVC also have a long timescale associated with the syllable repetition rate, reflecting stereotyped HVC activation for repeated syllables. For these reasons, we model the telencephalic input into the driven network as a simple periodic pattern (see top of Figure 1A). This simplified pattern corresponds then to the lowest frequency present in the Fourier expansion of the signal representing HVC activity when syllables are being repeated. We next introduce two neuron populations, one inhibitory and one excitatory, which are located downstream of HVC in the neural circuitry of the motor pathway. They constitute a minimal neural architecture capable of generating the second, driven timescale of our model. A schematic representation of this circuitry is shown in Figure 1A, and detailed equations and parameters of computational use can be found in Materials and Methods below. With this minimal model we can explore the effect of cooling by changing only two parameters: the frequency and amplitude of the forcing (i.e., HVC output). For example, with the HVC forcing frequency of 16 Hz (Figure 1A) the output of our model indicates a respiratory frequency of 8 Hz, which is a subharmonic solution (half the frequency of the forcing signal). The occurrence of subharmonic solutions is a clear signature of nonlinear processing of the input signal by the respiratory network and indicates interactive processing of the timescale of the input signal by the respiratory network resulting in a different, but subharmonically related timescale in the behavioral output (respiratory pressure gives rise to temporal sequencing of song).

Mild cooling is likely to result in a decrease in the forcing frequency. As long as respiration follows the forcing frequency in a 2∶1 (or 1∶1) locking, a simple stretching of the respiratory pattern results from the decrease in forcing (Figure 1B or 1C). However, when the forcing frequency changes further, presumably as a result of further cooling, the respiratory output frequency changes dramatically and predictably. A bifurcation takes place and manifests itself in a different locking of the forcing frequency and the respiratory output frequency (“breaking”). These simulations therefore show that stretching of syllables and their underlying respiratory gestures is consistent with an interactive model and therefore does not unambiguously indicate that telencephalic input dictates all timescales of the song [3], [16]. In addition, the model makes a strong prediction that further cooling will result in a predictable restructuring of the respiratory rhythm. Specifically “breaking” of syllables should occur and be visible in the fragmentation of complex syllables and their underlying respiratory patterns.

### Cooling of HVC: the breaking of syllables

**Figure 2:**
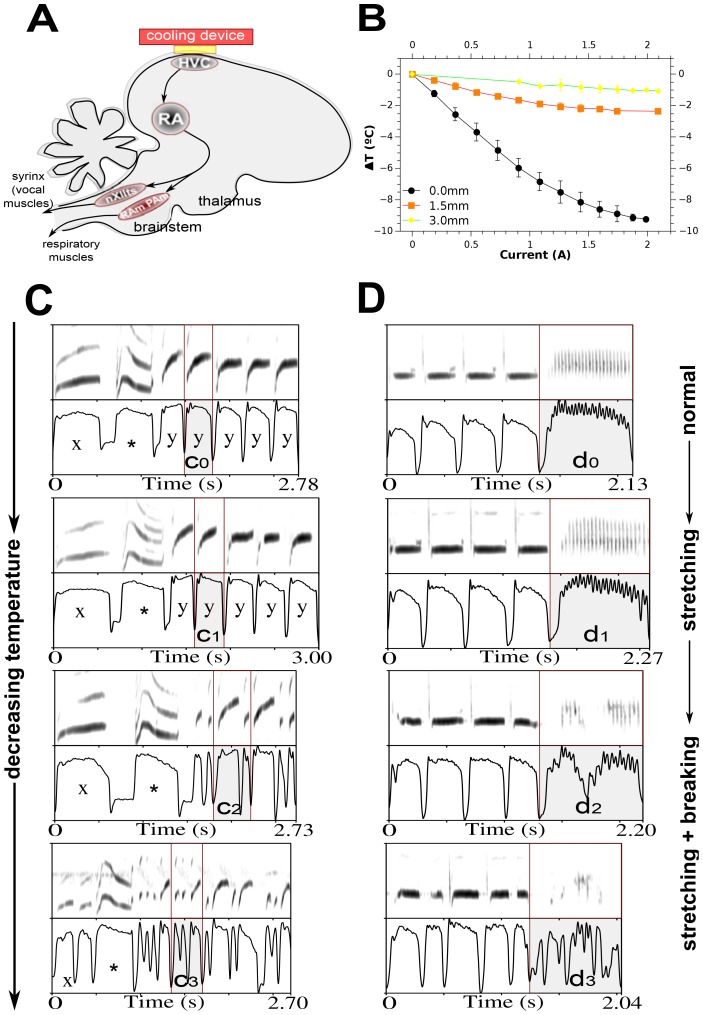
Experimental cooling of HVC. (A) Schematic of simplified song motor pathway with cooling device. HVC, used as proper name; RA, robust nucleus of the arcopallium; nXIIts, tracheosyringeal part of the hypoglossal nucleus; expiratory premotor nucleus RAm, nucleus retroambigualis; inspiratory premotor nucleus PAm, nucleus parambigualis. The cool side of the cooling device is positioned right against the dura over HVC. (B) Calibration of the cooling device showing brain temperature change as a function of the current applied to the device. Temperature measurements at different depths below HVC show that cooling is fairly local (n = 2). (C) Syllable breaking observed in bird #31. Shaded syllable first stretches and then breaks. Durations are *c_0_* = 320 ms, *c_1_* = 334 ms, *c_2_* = 428 ms and *c_3_* = 353 ms. Syllables have a different “breaking” point, which can be seen in the third and fourth row: syllable marked with an asterisk (*) does not break within this range of temperatures, and stops stretching in the third row, syllable marked with an “x” breaks at fourth row, and remaining syllables break at third row. Syllable frequencies range from 3 Hz in the first panel to 12 Hz in last panel. HVC temperatures from top to bottom are: normal, −2.6°C, −4.7°C and −7.5°C. (D) Syllable breaking observed in bird #37. Shaded syllable first stretches and then breaks. Durations are *d_0_* = 807 ms, *d_1_* = 870 ms, *d_2_* = 896 ms and *d_3_* = 793 ms. In the third row a deep pressure modulation arises and in the fourth row the syllable is broken into multiple expiratory pressure pulses. Pressure fluctuation, or syllable frequencies during the expiration are 34 Hz, 31 Hz and 28 Hz for first three rows. In the fourth row no clear sustained pattern exists, but in the segments with syllables they occur at 27 Hz. The song segment unmarked by shading stretches and then breaks in the fourth row. The corresponding HVC temperatures are from top to bottom: normal, −3.4°C, −4.8°C and −5.5°C. The total duration of the song segment indicates clear stretching of all patterns at first (second rows in (C) and (D)). Different syllables “break” at different temperatures (third and fourth rows), and the total duration of bout segments decreases after breaking occurs. Panel pairs in each row show spectrogram on top and recorded subsyringeal air sac pressure at bottom. Frequency range is 1–7 kHz. Pressure range is 0–1 in arbitrary units.

**Figure 3:**
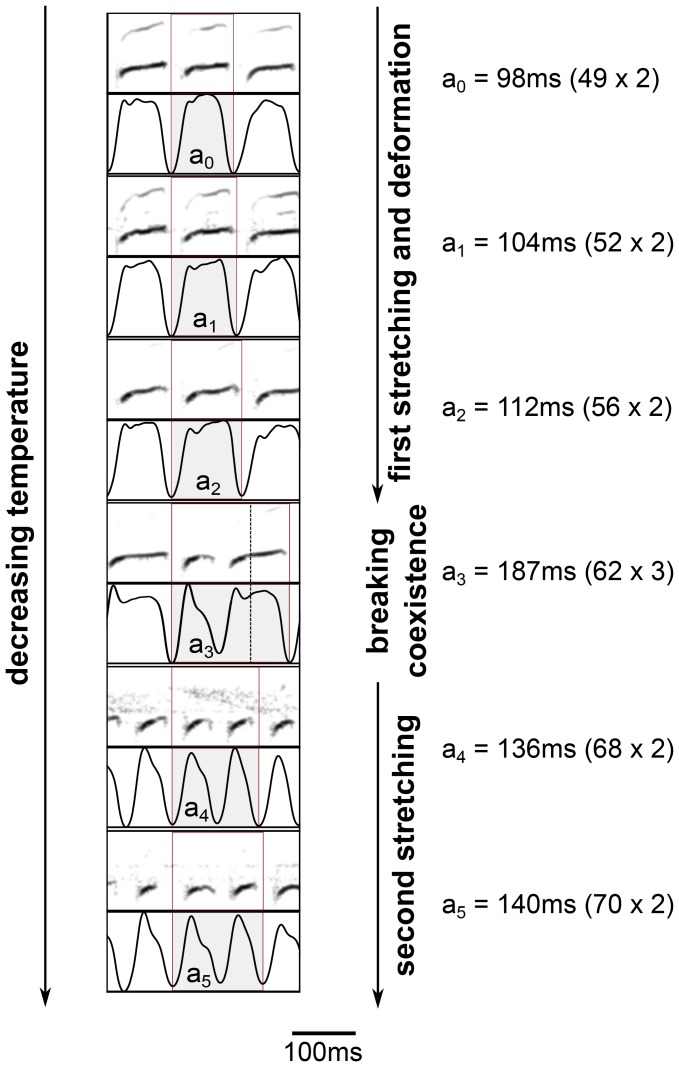
Cooling induces syllable stretching, deformation, and then syllable “breaking”. Example of song from canary #31 with the following HVC temperatures: normal, −2.6°C, −4.7°C, −5.4°C, −6.6°C and −7.5°C. Stretching occurs in the top three paired panels and is accompanied by a gradual change in the morphology of the pressure pulses and accompanying sound. The onset of breaking with coexistence of the broken and “unbroken”, deformed syllables is depicted in panel 4. The broken syllables get stretched upon further cooling (panels 5 and 6). The duration of the shaded syllables is: *a*_0_ 98 ms (49×2), *a*_1_ = 104 ms (52×2), *a*_2_ = 112 ms (56×2), *a*_3_ = 187 ms (62×3), *a*_4_ = 136 ms (68×2), *a*_5_ = 140 ms (70×2). For syllables *a*_i_ duration increments are evident as the duration increases from 49–52–56–62–68–70 ms. These durations correspond to half the syllable duration for i = 0, 1 and 2, one third of the combination of coexisting long and short syllable for i = 3, and a complete syllable for i = 4 and 5. These durations represent the period of the putative periodic instruction coming from HVC (see model in main text). If put together in a linear regression, results show that the largest stretch is of 45+−10%, and that the onset of breaking occurs at 33+−7%. Dashed line in *a*_3_ is at two thirds of its duration. Paired panels show the spectrogram (1–7 kHz frequency range) on top and subsyringeal air sac pressure on the bottom (0–1 in arbitrary units).

**Figure 4:**
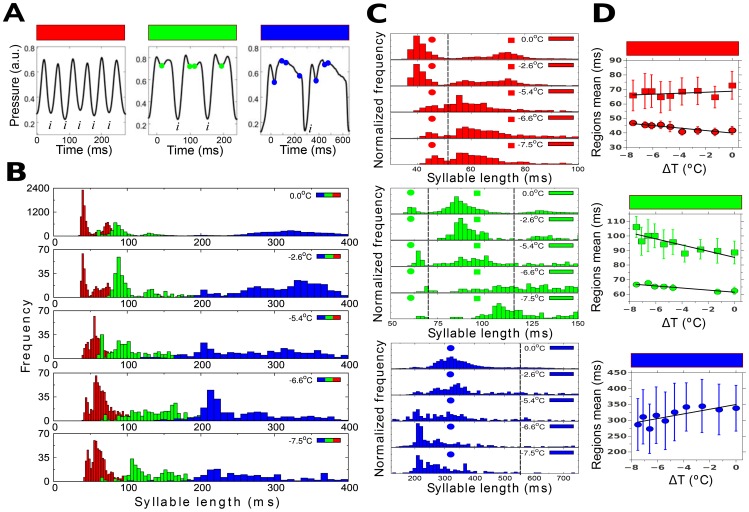
HVC cooling produces complex changes in the distribution of syllable duration. (A) Classification of syllables into three groups, depending on the number of local minima between two consecutive inspirations, denoted by “*i*”. In red we define syllables that are reminiscent to harmonic oscillations, with no minima. In this example there are six syllables separated by five inspirations. Syllables that have one or two local minima (green circles) are distinguished from those with three or more local minima (blue circles); three and two syllables are shown, respectively. The total number of syllables analyzed for this bird is 27471 (15040 red, 5830 green and 6601 blue). (B) Histograms of durations for every syllable recorded from bird #31 for different HVC temperatures. The bin size is 2.27 ms for red syllables 4.54 ms for green syllables and 9.07 ms for blue syllables. HVC temperature decreases from top to bottom (C) Individual normalized histograms of different types of syllables. Vertical dashed lines separate regions for computing statistical quantities. Bimodal red and green distributions are separated by their intermediate lowest frequency bin count, and for the remaining range limits we selected a region centered around the mean with three ssd width to each side. Distributions do not only drift to the right as expected from a stretching phenomenon, but relative quantities also change drastically due to syllable breaking and changes in the structure of the song. Colored circles and squares are used to label each region. (D) Variations with temperature of mean syllable duration distributions for regions in (C) (error bars are ssd). One red (squares) and one blue distribution indicate a slope opposite to that expected from stretching. The slopes of the linear regressions are as follows in ms/°C: red circles −0.86+−0.34, red squares 0.3+−1.4, green circles −0.71+−0.25, green squares −2.1+−1.1 and blue circles 7.4+−1.0.

In order to test these predictions, we cooled the left and right HVC in canaries with a Peltier cooling device [3] (Figure 2, A-B). In 7 males we simultaneously recorded subsyringeal air sac pressure and song at various temperatures of HVC. Cooling led to stretching of all pressure patterns and song syllables for a temperature decrease of up to 5°C (ΔT = −5°C). (Figures 2–4; S3–S6). If HVC was cooled further, however, many complex pressure pulses and associated syllables “break” into shorter segments.

Syllables shown in Figure 2 illustrate how, with increased cooling, air sac pressure pulses and song syllables are broken into shorter fragments. In Figure 2C, the song segment consists of two long syllables (labeled with × and *) followed by repeated upsweep notes. As the temperature is decreased, the pressure patterns and syllables of this latter type are first stretched (second panel) and then start to break into shorter patterns. Breaking of different syllables occurred at different temperatures. For example, the first syllable (x) of the song segment (Figure 2C) “broke” into two halves at a lower temperature than the upsweep notes, whereas the second syllable in the segment (*) did not “break” within the explored temperature range. A second example from a different individual (Figure 2D) illustrates the same effects for different syllable types. Repeated syllables, each arising from a distinct expiratory pressure pulse, are followed by a series of syllables generated by a sustained expiratory pressure pulse with pressure modulation at the syllabic rate (pulsatile syllable). Again, after initial stretching of air sac pressure pulses, the pulsatile syllable begins to “break” which manifests itself initially as a change in respiratory modulation and then in the occurrence of individual expiratory pulses separated by short inspirations.

In Figure 3 we display the restructuring of a different syllable from canary #31 starting with normal temperature, going through a transition and finally reaching a regular broken pattern as the temperature is decreased. This new pattern further stretches with decreasing temperature. At the transition temperature, where the onset of “breaking” occurs, there is a mixture of broken and stretched syllables, the duration of the former being approximately half that of the stretched syllables. As shown before, syllables stretch and then break, but this example also shows a breaking into a new regular pattern, whose timescale is consistent with a new locking regime following a transition period. Comparison with patterns of Figure 2C-D shows that “breaking” leads to a new regular pattern upon further cooling. Whereas syllables displayed there did not leave this transition regime, a new stable syllable duration is reached for the syllables of Figure 3 and this new duration is approximately half that of the original syllable. The “breaking” of syllables was observed in 4 out of 7 canaries and disappeared again when the temperature of HVC was allowed to return to body temperature. All 7 individuals displayed stretching of song syllables. Interestingly, in those individuals in which “breaking” was not observed HVC was not cooled by more than 5°C.

### Restructuring of syllable statistics

In order to assess whether these qualitative observations accurately represent the effects of cooling, we quantified syllable durations for all recorded song for each bird. We classified the syllables into three groups using the shape of the pressure patterns. This classification is based on the number of local minima found between two consecutive inspirations, which define the onset and offset of each syllable (See Figure 4A, and Materials and Methods below). The first group corresponds to patterns without local minima, the second group to patterns with one or two local minima, and the third group contains patterns with three or more local minima. We then compared histograms with the durations of each syllable category as displayed for one individual in Figure 4B-D. Syllable stretching is expected to be associated with distributions of syllabic durations that are displaced to the right (longer durations) and do not change in shape. Changes in the shape of the distributions can be caused either by a change in the occurrence of the syllable types, or by the breaking of pressure pulses and syllables into shorter segments that get classified differently. All of these effects are found in our data, supporting our qualitative observations. Figure 4C displays the distributions of syllabic durations of each group, as HVC is increasingly cooled from ΔT = 0 to −7.5°C. In this case, the distributions of syllabic durations of the first group (red) and second group (green) are bimodal. As the temperature decreases, the first mode moves to the right. The same effect is observed for the second mode of the second group (green). In contrast, syllables forming the second mode of the first category (red) and the entire distribution for the third category (blue) increase in frequency with increased cooling and new modes arise at shorter syllable duration. To quantify these effects we computed the mean and sample standard deviation (ssd) for fixed ranges of syllabic lengths found at each cooling value. These ranges were selected based on the analysis of the distributions obtained without cooling. We separated bimodal red and green distributions by their intermediate lowest frequency bin count (dashed line in Figure 4C), and for the remaining range limits we selected a region centered around the mean with three ssd width to each side. These mean values show changes across the range of cooling, with negative slopes indicating a stretching effect. As expected, some ranges for syllable duration show the stretching response with decreasing temperature (see Figure 4D). Two ranges of syllable duration show mean values that increase with decreasing temperature, suggesting a second effect of cooling. Stretching percentages are underestimated by these calculations because the regions are fixed and contain different syllables of the same group. Stretching cannot explain the positive slopes, but these indicate a restructuring of some syllables, consistent with “breaking”. Additional analyses of individual syllables are presented in Figures S5–S6.

### Bifurcation diagram of the model

**Figure 5:**
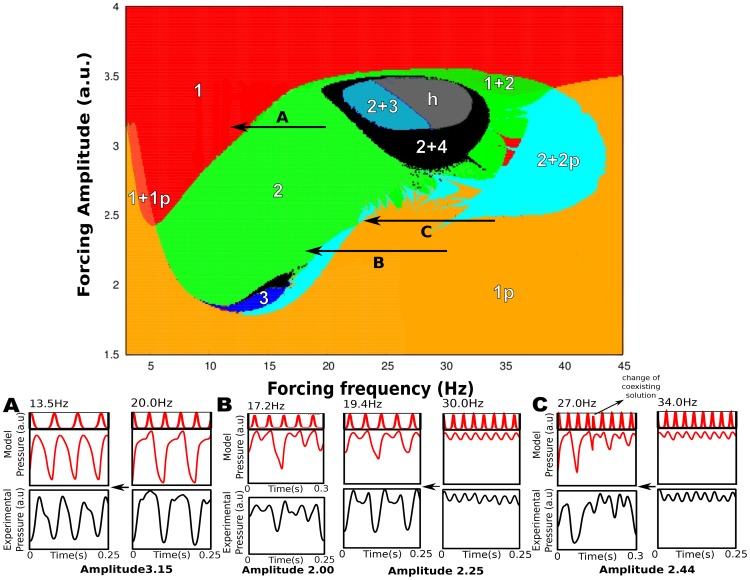
Bifurcation map of the model predicts different types of breaking. Each coordinate of the map corresponds to a pair of parameters *(f,A)* These represent the frequency and amplitude of the forcing in our model. Colored (and numbered) regions correspond to different locking regimes between the forcing frequency and the output of the model. Regions with “p” (pulsatile) labels are for output patterns with oscillations on top of a constant value (long expiration). The “+” sign is used to denote coexistence of solutions. Color code (numeration) is as follows. Red (1) corresponds to a 1∶1 locking between the forcing and the output of the model. In the region colored with green (2) there is a 2∶1 locking. This means that the output pattern will repeat itself after a time equal to twice the forcing period. The region colored with blue (3) denotes a 3∶1 locking (i.e., the output repeats itself after a time equal to three times the forcing period). The orange region presents pulsatile solutions (1p), which are locked 1∶1 with the forcing. The light red region (1+1p) gives rise to either pulsatile or harmonic looking solutions depending on the initial conditions, both locked 1∶1 to the forcing. The region colored with cyan (2+2p) gives rise to solutions of 2∶1 locking. The other colors denote regions of the parameter space with solutions in other locking regimes. Cooling is associated with horizontal arrows A-C pointing to the left (decreasing frequency), and the breaking of syllables is interpreted as bifurcation transitions between the different regions. We mapped three syllables where we found breaking from normal and cooled song, to the beginning and end of arrows respectively. (A) Syllable (same as Figure 3, canary #31) originally sung in regime 2 at 10 Hz (*f* = 20 Hz) that ends at region 1 at 13.5 Hz., both with *A* = 3.15 (a.u.). (B) Syllable in regime 1p (orange colored) at 30 Hz of canary #37 crosses border to end at region 2 at 9.7 Hz (*f* = 19.4 Hz), with *A* = 2.25. In this case, at the coldest temperature, there is an experimental pattern that we matched with locking regime 3 (blue colored), which is very close at *f* = 17.2 Hz and A  = 2.0 (a.u). (C) Syllable from region 1p (same as Figure 2D, canary #37) that crosses to region 2+2p (coexistence of period 2, and period 2 above a constant expiration). The cold temperature pattern shows a lack of repetitive syllable structure that can be explained with the coexistence of solutions of our model. We used two different initial conditions that we changed in the middle of the simulation, resulting in a strong resemblance with the experimental pattern. All mentioned border crossings are different bifurcations of our model that are manifested in the cooling experiment and show its predictive capability for a wide parameter range. Model pressure has on top a scaled pattern of HVC activity to visualize the locking regime. Pressure is 0–1 in arbitrary units.

The changes to syllable duration distributions were consistent with our dynamical model in that they exhibited both a predicted stretching of some syllables and a more complex “breaking” and restructuring of other syllables. Moreover, detailed changes to specific identified syllables that could be followed over the course of cooling, were consistent with expectations from the model. For example, Figure 5 shows a representation of expected restructuring/bifurcation of syllable structure predicted as a function of changes to driving frequency and amplitude.

In our model, the driving frequency corresponds to a low component in the Fourier expansion of the activity of the forcing signal, which we expect to be present in the cases where repeated syllables occur. We assume that the simplest pressure pattern, which looks as a harmonic signal, is the result of a 1∶1 locking between the driving and driven timescales. Therefore, the starting point of our analysis assumes a forcing frequency equal to the measured syllabic rate (see Figure 5, pattern A, left). For different values of the forcing parameters (frequency and amplitude), different dynamical regimes are expected. In Figure 5 (top), the colored regions are called “Arnold tongues” [20] in the language of dynamical systems theory. Within these “tongues” the solutions preserve their topological structure. In this paradigm, cooling of HVC corresponds to decreasing the forcing frequency, i.e. a horizontal shift to the left in the parameter space. This is represented in the bifurcation diagram by arrows. We found that cooling induced changes to syllable structure/respiratory patterns that matched the predictions from this model.

Figure 5A-C shows three experimental and modeled realizations of syllables sung by canaries #31 and #37 at normal and cooled HVC. Corresponding horizontal arrows (A-C) on the map indicate the possible path in our model parameters resulting from the experimental decrease in HVC temperature. Figure 5A shows a syllable (same as Figure 3) originally sung in regime 2 (green, 2∶1 locking) that ends in region 1 (red, 1∶1 locking). In Figure 5B we found a syllable in regime 1p (orange, 1∶1 locked above a sustained expiration) that crosses the border into region 2. In this case, at the coldest temperature, we matched an experimental pattern with locking regime 3 (blue, 3∶1 locking). Finally, Figure 5C has a syllable from region 1p (same as Figure 2D) that crosses to region 2+2p (coexistence of 2∶1 locking, and 2∶1 above a constant expiration). The lack of a repetitive structure of this broken pattern is explained by the coexistence of solutions in our model. All these border crossings represent different bifurcations of the model that can be used to interpret the cooling experiment.

The quantitative results are consistent with the qualitative observations that syllables belonging to group three are most likely to be affected by “breaking”. Notice that this is only a necessary condition: syllables that break should not only be subharmonic but should also be close to the boundary of an Arnold Tongue. For example, long syllables with modulated sustained expiratory pressure (pulsatile pattern of Figure 2D) break into shorter expiratory pressure pulses and now get classified as multiple elements of shorter duration within groups 1–3, such that the longest elements of group 3 no longer occur. Typically, syllables of higher categories tend to break at higher temperatures than syllables belonging to category 1 (red), which are generally very similar to harmonic oscillations [12], [13]. Therefore, breaking of this nature leads to two effects for the quantitative analysis, (1) it increases the occurrence of groups one and two, and (2) it results in shortening of the mean duration for group three.

## Discussion

We present strong theoretical and experimental evidence that complex motor patterns underlying birdsong production are consistent with the nonlinear interaction between timescales of different components of the motor control network. Our minimal model predicts that a small change in the frequency of an average instruction coming from HVC may provoke a drastic change in the temporal pattern of resulting syllables and, thus, facilitates a control mechanism where simple neural instructions interacting with downstream neural architecture can result in complex rhythms in the output patterns. Manipulation of the telencephalic timescale through local cooling results in the predicted effects of initial stretching and then “breaking“ of syllables. These syllable “breaking” patterns can be interpreted in terms of bifurcations of the model. The bifurcation map of our model shows its predictive power for a wide range of parameters. Qualitatively different types of syllable breaking were found to match horizontal paths on this map. In addition, quantitative results for different syllables correspond to explanations derived from the model. Border crossing from region 2 to 1 turns syllables from group 2 into syllables of group 1. Pulsatile patterns from region 1p that belong to group 3 turn into shorter members of that group when crossing to region 3, or become members of group 2 when crossing to region 2 or 2+p.

More than one timescale is necessary to explain our results. In a nonlinear interaction paradigm, the first effect is indeed stretching as long as a specific locking relationship is maintained between the interacting timescales. Therefore, the stretching of zebra finch song can be interpreted as a nonlinear locking effect, rather than as the result of a look up table linking bursts of HVC activity with brief segments of song [3], [18], [21], [22]. This challenges a widely accepted view according to which HVC bursts determine a unique timescale in the birdsong system [3]. Combining the results from this study and those obtained in zebra finches, it is most parsimonious to postulate an interactive mechanism in all songbirds. We do not propose a specific location for the second timescale necessary to explain the shape and rate of the measured pressure patterns and their breaking. A minimal mathematical implementation consists of an excitatory and inhibitory population, and even at the scale of one nucleus this structure is possible (e.g., nucleus RA). Other architectures provide a plausible substrate for the emergence of this second frequency, as the one proposed in [11], or the integrative circuit describing the interaction between forebrain and brainstem nuclei described in [23]. The results reported for zebra finches represent the expected changes of initial cooling, but temperatures where “breaking” might occur may not have been reached in these studies. The interactive model proposed here may represent a more general mechanism that enables generation of highly complex motor patterns with relatively simple telencephalic instructions.

In conclusion, if this alternative, interactive model for how different timescales may be generated in the respiratory pattern of birdsong is correct, it requires a rethinking of the specific role of HVC in song motor control. In this model at least two nonlinearly interacting time and spatially separated timescales are present. Their qualitatively different output rhythms give rise to different respiratory patterns of song. Importantly, this model is completely consistent with the observed firing patterns of RA-projecting neurons [16], [17] and the results of the cooling experiments in zebra finches [3], [18].

Song motor control requires the integration of respiratory muscles and the muscles of the two independently controlled sound generators of the avian vocal organ, the syrinx. Syringeal control contributes to the very short timescales of song features (e.g., onset of vibrations, rapid frequency and amplitude modulation, etc.), whereas the syllable repetition rate and slower amplitude modulation can arise from modulation of respiratory pressure [1], [24], [25]. It is not known whether the specific output patterns from HVC to respiratory and syringeal projection areas within RA differ or whether the downstream circuitry generates respiratory and syrinx specific control patterns from similar input signals. A recent proposal that RA-projecting neurons in zebra finches specifically relates to transition states (acoustic and respiratory) [26] supports theoretical work that proposes an integrated, non-linear mechanism for syringeal motor control [27]. Although further experimental and theoretical work is needed to illuminate syringeal motor control mechanisms, these studies also challenge the notion that HVC directly controls all timescales of song.

## Materials and Methods

### Ethics statement

All experiments were approved by the Institutional Animal Care and Use Committee of the University of Utah and of the University of Buenos Aires.

### Subjects: training and procedures

Male canaries were acquired from a local breeder. Vigorously singing birds were subjected to sequential implantation of cooling and recording devices as described below. In total, a complete data set was collected from 7 birds. For surgeries birds were anesthetized with a mixture of ketamine and midazolam, and deprived from food 40 minutes prior to each surgery. First we attached a helmet for gradual weight training and future positioning of the cooling device. The skull of the birds was exposed by an incision of the skin, and a ceramic helmet (0.3 g) was attached with dental cement (Dentsply, Tylok Plus). The midsagittal bifurcation or zero point was marked. Birds were accustomed to added weight by increasing the helmet mass by 0.5 g every other day until a final mass of 2.0 g was reached. A training backpack (2.0 g) with the same connectors as those on the cooling device was placed on the back of the bird. After reaching the final weight of 2.0 g on the head, they were trained for 4 hours for at least 2 days with a thick silastic tubing coming from top of the cage, connected to the backpack. Next the cooling device was attached by making two openings in the skull (1.5 mm×2.0 mm) in the regions over both HVC. The gold implant was then placed on the dura and the cooling device was pasted to the helmet of the bird with glue adhesive and contact adhesive. A cooling device can be seen in Figure S1C. and the device mounted on a bird in Figure S1D. The mass of the device was 1.7 g on the head, and 2.0 g on the backpack. We also inserted a cannula (Dow Corning, Silastic Laboratory Tubing NO. 508-005) into the anterior-thoracic air sac to measure subsyringeal air sac pressure as described in [24]. This procedure was performed at least three days after the attachment of the cooling device and after the birds resumed singing. The free end of the cannula is connected to a miniature piezoresistive pressure transducer (Fujikura model FPM-02PG) that was mounted on the bird's backpack.

### Temperature measurements

Six different cooling devices were used in this experiment. A Peltier module works by providing a constant difference in temperature for each driving current, for a constant temperature in its hot side. If the hot side is kept at a constant temperature, the cool side varies its temperature in an almost linear relationship with the current applied as shown in Figure 1B. A mainly local reduction in temperature of almost 10°C for the cool side is achieved with approximately 2A. The temperature of the hot side of the device was kept at room temperature. Calibrations were made right after the device was attached, to account for differences between the different cooling devices (Figure S2).

### Song and pressure measurements

Birds were placed in tall cages of 21 cm×34 cm base and 36 cm height in an acoustic chamber and connected for water circulation, power supply and pressure signal acquisition. A microphone (TAK-STAR SGC 568) was placed in front of the cage. Sound and pressure signals were recorded using a multichannel sound card (MAYA 1010, 44.1 kHz sample rate) and fed directly to the computer with custom MATLAB software. The pressure signal was preamplified and modulated (multiplied by a sinusoidal at a constant frequency around 2 kHz) to fit sound card requirements. For analysis, the pressure signal was demodulated and resampled at 882 Hz, i.e., the envelope of the recorded signal, which is the actual pressure pattern, was obtained with a zero-phase digital filtering and then downsampled. Measurements consisted of recordings of song and pressure without cooling first. Then we lowered the temperature by 1C° every two minutes until the desired temperature was reached and, after two minutes at the respective temperature, recording was started. Once sufficient data were collected at a given temperature, we proceeded with further cooling. Because spontaneous song delivery varies, we recorded song for 1–4 different temperatures per day. After each cooling episode, we recorded control data when the temperature had returned to body temperature.

### Statistical analysis of pressure patterns

Simultaneous recordings of audio and pressure signals were triggered automatically with custom MATLAB software and lasted from 2 seconds to 2 minutes. Custom software (C) was written to recognize song syllables from pressure recordings. The average (*avg*), the maximum (*max*) and the minimum (*min*) pressure value in each file was computed. Local minima were found, and the ones with values over a threshold of *avg-0.04(max-min)*, corresponded to local minima inside syllables. Quiet respiration was successfully discarded with these two criteria: 1) maximum pressure between two consecutive minima is below *0.5avg+0.29(max-min)*; 2) total points between two consecutive minima have less than 32% data points over *avg*. Finally, computing the area under each putative syllable and discarding the ones below a threshold eliminated short fluctuations due to noise. Remaining consecutive minima, denote beginning and end of syllables. Syllable durations were computed for construction of histograms for the different measured temperatures for each bird.

### Individual syllables

The study of broken syllable motifs along different cooling temperatures showed a drastic change in morphology and syllable duration. For their proper recognition, every song sequence of the canaries studied was labeled by inspection, and different syllables were recognized from their vocalization frequencies and their position in similar song sequences, since, generally, many syllables only stretched.

### Modeling details

We modeled average instructions coming from HVC with a rhythmic period *T*, since canary song is formed of phrases in which syllables are repeated up to dozens of times. Each period of the forcing instruction of period is defined as follows in 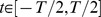:
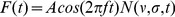
(1)where *A* is the amplitude, *f = 1/T* is the frequency of the instruction and *N(ν,σ,t)* is the normal distribution, centered at *ν = 0*, with a width *σ* of 9.5% of the period *T*.

For the circuitry downstream of HVC we built our model introducing the minimal neural populations necessary for an oscillatory behavior: 2 [20]. Equations for average activity of neuron populations *x*, excitatory, and *y*, inhibitory, are as follows: 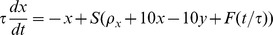


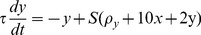
(2)where 
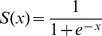
 is a soft step function whose arguments in the equations represent the input activity for each neuron population. The parameter *ρ* represents basal input activity and *τ* is a scaling constant. Numerical values in the equation are the same as in [28]. Values for *ρ*_x_ and *ρ_y_* were selected for the system to be near a Takens Bogdanov bifurcation and have patterns similar to those found in canaries: *ρ*_x_ *= −2.8* and *ρ*_y_ *= −10.7* [12]. The parameter *τ = 0.0088* was selected to match our simulated pattern frequencies with the ones measured in the canaries.

Exploration of parameter space *(f,A)* showed the behavior of stretching and breaking (bifurcation transition), with a close morphology and syllable rate match between the experimental measurements and simulated pressure patterns.

### Bifurcation diagram

For a given coordinate *(f,A)* of the forcing HVC frequency of the model, we performed simulations of the pressure patterns for a set of 121 initial conditions in the range [0∶1] for *x* and *y*, and allowed to reach a stationary solution after approximately 10 periods *T*. Then we computed a Poincaré Map at different multiples of *T* to find the locking regimes [20]. Pulsatile regimes were recognized having an average solution above 0.8. Coexistence of solutions were confirmed by plotting all the stationary solutions in a 3 dimensional plot with coordinates *(t,x,y)* that revealed the orbits topological organization.

In order to fill the diagram, we performed steps of 0.01 in *A* and 0.1 in *f* and computed the locking regime. We explored a range of parameters where we found wide diverse syllable patterns and frequencies that match the ones measured in the experiment, assigned a color to each locking regime and plotted the map with this code.

## Supporting Information

Figure S1 **The cooling device.** (**A**) Top and side view of a Peltier piece cut from a commercial Thermoelectric Cooler (HB corporation, TEC1-12706) with a Low Speed Diamond Wheel Saw. Its dimensions are 1 cm×1 cm with 4 mm height in thicker part (0.7 g). Only four semiconductor elements were kept and a thin ceramic region for the cool side. Thin wires (Alpha Wire Company, multicolor 4 wires AWG 32) were soldered for the current supply (0.2 g). (**B**) Top left: Water chamber made with hot melt adhesive poured on a mold with tubes and an aluminum base (1.0 g). Bottom left: top view of chamber where two openings are left for tubes of 6 cm and 0.3 g (Dow Corning, Silastic Laboratory Tubing NO. 508-005). Top right: chamber with tubes and without base. Sealant (AKAPOL SA, Fastix) is used to prevent water leakage. Bottom right: the base of the chamber is an aluminum piece cut from common electronic cooling components of size 1 cm×1 cm, 1.7 mm height and 0.4 g weight. (**C**) Complete device (3.7 g). Chamber and Peltier are attached with general purpose thermally conductive epoxy (Cytec, Easypoxy K20). At the end of wires we soldered a miniature simple row connector (0.3 g). Pipe connectors from common aquarium tubing are glued to the end of the tubes (0.4 g). The pressure sensor (Fujikura; 0.7 g) has two pairs of connectors for the pressure signal and its power supply. A bronze, nickel-gold plated implant is attached to Peltier's cool side (0.2 g). Its base is 1.5 mm×6.8 mm and inclined contact pads (25°) have a base of 1.5 mm×2 mm with a separation of 2.8 mm. (**D**) Freely behaving canary with the device on it. The backpack allowed more freedom of movement.
(EPS)

Figure S2 **Temperature calibrations of dura surface for the six cooling devices of our experiment right after implantation.** Previous calibrations with the same devices in unsuccessful subjects that did not sing are included. Calibrations were divided in two groups: the devices that were used by birds that broke syllables (N = 12) and the ones used by the birds that only stretched their song (N = 10). Points are mean and error bars are ssd. There is a noticeable difference in the slope of temperature decrease for these two groups: (−5.5+−0.8)°C/A and (−4.4+−1.1)°C/A. Calibrations were made at half scale (1 A) for better recovery of the birds after surgery. Installation of device lowers brain temperature by approx. 1°C at 0 A, as noted in [18], because of the water reservoir at room temperature. Coolest temperatures reported in main article are obtained by extrapolation of each animal calibration, without this first temperature drift.
(EPS)

Figure S3 **Histograms of syllable duration at different HVC temperatures of three canaries that showed broken syllables.** Syllables were divided into three groups depending on the number of local minima between two consecutive inspirations. In red we define syllables that are reminiscent of harmonic oscillations, with no minima (first column). Green is used for syllables that have one or two local minima (second column). Blue is used for syllables with three or more local minima (third column). Most distributions only drift to the right since syllables get longer with decreasing temperature. (**A**) In canary #32 the right part of the green distribution first shifts to the right and is then depleted with more cooling. Total syllables analyzed are 24760 red, 9148 green and 7327 blue. (**B**) Canary #37 shows a right bimodal blue distribution first drift to the right and then a depletion. Total syllables analyzed are 3280 red, 1175 green and 1053 blue. (**C**) In canary #49 the right part of the green distribution first shifts to the right and is depleted upon further cooling. Total syllables analyzed are 2486 red, 2563 green and 2306 blue.
(EPS)

Figure S4 **Histograms of syllable duration at different HVC temperatures for three canaries that did not show broken syllables.** Syllables are divided into 3 groups as specified in Figure S3. Distributions only shift to the right since syllables only get longer with decreasing temperature. (**A**) Canary #28. Total syllables analyzed are 4161 red, 1033 green and 835 blue. (**B**) Canary #29. Total syllables analyzed are 38439 red, 20387 green and 7464 blue. Second mode of green distribution at −3.2°C shifts with increased occurrence of syllables below 125 ms. (**C**) Canary #50. Total syllables analyzed are 4293 red, 5006 green and 3906 blue. Red group at −3.9°C lacks the first mode of the distribution. Songs did not contain syllables around 50 ms in length at this temperature.
(EPS)

Figure S5 **Individual statistics for broken syllable “x”, as labeled in** **Figure 2C** **.** This common syllable presents a pattern that could be followed across temperatures. (A) Paired panels of sonogram (top, 1–7 kHz) and pressure gestures (bottom, 0–1 a.u.) present on top the syllable at normal temperature and below, the syllable found at −4.7°C. The syllable breaks into one half and two quarter parts, and the addition of minibreaths results in a slight increase in total length. (B) Histograms showing the evolution of this syllable's classification and duration across temperatures, where there was enough information to compute statistics (see Figure 3A for color code). Syllable duration is not constant at normal temperature, but has a distribution centered around 600 ms with a range from 500 ms to 700 ms. At −4.7°C, the distribution breaks, and blue classified syllables have a new mode centered at around half the length of the original one. Green classified syllables spread at half the duration of this new mode. Long-duration syllables occur much less frequently; we do not find syllables above 710 ms at −4.7°C, and above 300 ms for the new mode at −7.5°C. (C) Stacked columns show the percentage of syllable types at corresponding temperatures. In cyan we denote syllables classified as blue, but below 400 ms in length. Breaking at −4.7°C affects only half of the syllables, and at −7.5°C, it affects an 83% of them. In addition, broken syllables of quarter length of the original have more incidents at lower temperatures, reaching 33%. (D) Quantification of distribution means and standard deviation from (B). At −4.7°C the cyan and green distributions have a mean value that is approximately one half and one quarter the mean presented by the blue distribution. At −7.5°C there are only three syllables found above 400 ms and no values were computed. The mean of cyan distribution decreases as syllables above 300 ms occur less frequently.
(EPS)

Figure S6 **Individual statistics for broken syllables labeled with “y” as in** **Figure 2C** **.** These frequently sung syllables present a pattern that could be followed across temperatures. Although they could be further classified in two types (shaded and previous syllable in Figure 2C are slightly different with respect to the three syllables following them), this classification was not always possible when the patterns were broken, and all of them were considered for the quantification. (A) Paired panels of sonogram (top, 1–7 kHz) an pressure gestures (bottom, 0–1 a.u.) present on top syllables at normal temperature, in the middle at −4.7°C and at the bottom at −7.5°C. Syllables break into half and quarters, and have an increase in total length. (B) Histograms showing the evolution of this syllable's classification and length across temperatures (see Figure 3A for color code). Syllable length is not constant at normal temperature, but it has a distribution centered at around 310 ms with a spread that goes as far as 400 ms and 250 ms. At −4.7°C, the distribution starts to break and the syllables classified as blue have a new green mode centered at around half the length of the blue ones. Red classified syllables spread at half length of this new mode. (C) Stacked columns show the percentage of syllable types at the corresponding temperatures. Breaking affects an increasing percentage of syllables as the temperature decreases and these change group when broken. (D) Quantification of distribution means and standard deviation from (B). The dotted line corresponds to a linear fit for the means of the blue syllables before breaking takes place. Below −4.7°C a linear extrapolation with circles was made to show the expected value of the mean of the blue type if the syllables were not broken. The green and the red distributions have a mean value that is approximately one half and one quarter the one of the extrapolated value. Computed mean of blue distribution decreases due to its depletion above 310 ms.
(EPS)

## References

[1] Zeigler HP, Marler P (2008) Neuroscience of birdsong. (Cambridge,Cambridge University Press ).

[2] Méndez JM, Mindlin GB, Goller F (2012) Interaction between telencephalic signals and respiratory dynamics in songbirds. J Neurophysiol 107: 2971–2983. 22402649 10.1152/jn.00646.2011 PMC3378361

[3] Long MA, Fee MS (2008) Using temperature to analyse temporal dynamics in the songbird motor pathway. Nature 456: 189–194. 19005546 10.1038/nature07448 PMC2723166

[4] Smotherman M, Kobayasi K, Ma J, Zhang S, Metzner W (2006) A mechanism for vocal-respiratory coupling in the mammalian parabrachial nucleus. J Neurosci 26: 4860–4869. 16672660 10.1523/JNEUROSCI.4607-05.2006 PMC6674146

[5] Ahrens KF, Kleinfeld D (2004) Current Flow in Vibrissa Motor Cortex Can Phase-Lock With Exploratory Rhythmic Whisking in Rat. J Neurophysiol 92: 1700–1707. 15331651 10.1152/jn.00020.2004

[6] Berg RW, Kleinfeld D (2003) Vibrissa Movement Elicited by Rhythmic Electrical Microstimulation to Motor Cortex in the Aroused Rat Mimics Exploratory Whisking. J Neurophysiol 90: 2950–2963. 12904336 10.1152/jn.00511.2003

[7] Kakei S, Hoffman DS, Strick PL (1999) Muscle and Movement Representations in the Primary Motor Cortex. Science 285: 2136–2139. 10497133 10.1126/science.285.5436.2136

[8] Friedman WA, Jones LM, Kwegyir-Afful EE, Zeigler HP, Keller A, et al (2006) Anticipatory Activity of Motor Cortex in Relation to Rhythmic Whisking. J Neurophysiol 95: 1274–1277. 16251259 10.1152/jn.00945.2005 PMC1388275

[9] Friedman WA, Zeigler HP, Keller A (2011) Vibrissae motor cortex unit activity during whisking. J Neurophysiol 107: 551–563. 21994257 10.1152/jn.01132.2010 PMC3774649

[10] Cramer NP, Keller A (2006) Cortical control of whisking central pattern generator. J Neurophysiol 96: 209–217. 16641387 10.1152/jn.00071.2006 PMC1764853

[11] Trevisan MA, Mindlin GB, Goller F (2006) Nonlinear Model Predicts Diverse Respiratory Patterns of Birdsong. Phys Rev Lett 96: 058103. 16486997 10.1103/PhysRevLett.96.058103

[12] Alonso LM, Alliende JA, Goller F, Mindlin GB (2009) Low-dimensional dynamical model for the diversity of pressure patterns used in canary song. Phys Rev E 79: 041929. 10.1103/PhysRevE.79.041929 19518278

[13] Alliende JA, Méndez JM, Goller F, Mindlin GB (2010) Hormonal Acceleration of Song Development Illuminates Motor Control Mechanism in Canaries. J Develop Neurobiol 14: 943–60. 10.1002/dneu.20835 PMC2987553 20812319

[14] van der Pol B, van der Mark J (1927) Frequency Demultiplication. Nature 120: 363–364.

[15] Strogatz SH (2001) Nonlinear dynamics and chaos: with applications to physics, biology, chemistry, and engineering (studies in nonlinearity). Westview Press.

[16] Hahnloser RHR, Kozhevnikov AA, Fee MS (2002) An ultra-sparse code underlies the generation of neural sequences in a songbird. Nature 419: 65–70. 12214232 10.1038/nature00974

[17] Fee MS, Kozhevnikov AA, Hahnloser RHR (2004) Neural Mechanisms of Vocal Sequence Generation in the Songbird. Ann N Y Ac of Sc 1016, volume 1016: 153–170. 10.1196/annals.1298.022 15313774

[18] Andalman AS, Foerster JN, Fee MS (2011) Control of Vocal and Respiratory Patterns in Birdsong: Dissection of Forebrain and Brainstem Mechanisms Using Temperature. PloS One 6: e2546 doi:10.1371/journal.pone.0025461 10.1371/journal.pone.0025461 PMC3182229 21980466

[19] Prather JF, Peters S, Nowicki S, Mooney R (2008) Precise auditory-vocal mirroring in neurons for learned vocal communication. Nature 451: 305–310. 18202651 10.1038/nature06492

[20] Guckenheimer J, Holmes P (1997) Nonlinear oscillations, dynamical systems, and bifurcations of vector fields. Springer-Verlag.

[21] Schmidt MF, Ashmore RC, Vu ET (2004) Bilateral Control and Interhemispheric Coordination in the Avian Song Motor System. Ann N Y Ac of Sc 1016: 171–186. 10.1196/annals.1298.014 15313775

[22] Schmidt MF, Ashmore R (2008) Integrating breathing and singing: forebrain and brainstem mechanisms. In:Zeigler and Marler, eds Neuroscience of Birdsong, Neuroscience of Birdsong. (Cambridge, Cambridge University Press), pp 115–135.

[23] Ashmore RC, Wild M, Schmidt MF (2005) Brainstem and Forebrain Contributions to the Generation of Learned Motor Behaviors for Song. J Neurosci 14 25: 8543–8534. 10.1523/JNEUROSCI.1668-05.2005 PMC6725679 16162936

[24] Goller F, Suthers RA (1996) Role of syringeal muscles in controlling the phonology of bird song. J Neurophysiol 76: 287–300. 8836225 10.1152/jn.1996.76.1.287

[25] Mindlin G, Laje R (2005) The Physics of Birdsong. Springer.

[26] Amador A, Sanz Perl Y, Mindlin GB, Margoliash D (2013) Elemental gesture dynamics are encoded by song premotor cortical neurons. Nature 495: 59–64. 23446354 10.1038/nature11967 PMC3878432

[27] Trevisan MA, Mindlin GB (2007) The constraints to learning in birdsong. Eur Phys Journal 146: 199–204.

[28] Hoppensteadt FC, Izhikevich EM (1997) Weakly connected neural networks.Springer .

